# The presence of psychological distress in healthcare workers across different care settings in Windsor, Ontario, during the COVID-19 pandemic: A cross-sectional study

**DOI:** 10.3389/fpsyg.2022.960900

**Published:** 2022-08-29

**Authors:** Jennifer Voth, Lindsey Jaber, Linda MacDougall, Leslee Ward, Jennifer Cordeiro, Erica P. Miklas

**Affiliations:** ^1^Hôtel-Dieu Grace Healthcare, Windsor, ON, Canada; ^2^Faculty of Human Kinetics, University of Windsor, Windsor, ON, Canada; ^3^Faculty of Education, University of Windsor, Windsor, ON, Canada; ^4^ St Clair College, Chatham, ON, Canada

**Keywords:** healthcare workers, COVID-19, psychological distress, psychology, healthcare, mental health

## Abstract

**Introduction:**

Few studies have examined psychological distress in healthcare workers (HCWs) across the care continuum. This study describes distress levels reported by HCWs across care settings and factors associated with distress.

**Methods:**

A cross-sectional survey of HCWs from Windsor, Ontario, was conducted between May 30th, 2020, and June 30th, 2020. The survey included the Kessler Psychological Distress Scale (K10), sociodemographic, frontline status, perceptions of training, protection, support, respect among teams, and professional and personal stressors. Univariate analyses were used to compare across settings and multivariate logistic regression assessed factors associated with distress.

**Results:**

Four hundred and three HCWs from the hospital (49.4%), community health and social service (18.4%), first responder (14.7%), primary care (7.9%), home (6.0%), and long-term care (LTC; 4.0%) participated in the survey. Common concerns included fear of transmitting COVID-19 to family, safety on the job, and balancing personal care with work demands. LTC and home-care HCWs reported greater concern about workload and staffing levels, whereas community health workers were more anxious about their financial security. Overall, 228 (74.2%) HCWs who completed the K10 reported high distress, with greater rates among hospital and LTC HCWs. Distress was more likely in HCWs who identified as female, younger than 55, perceived lower respect among team, and experienced greater worry about physical and mental health and managing high workloads.

**Conclusion:**

Results showed a high degree of distress experienced by HCWs across care settings and the impact of the COVID-19 pandemic on personal and work-related stress. Promoting self-care and supportive and collaborative healthcare teams are promising avenues for mitigating symptoms of distress.

## Introduction

By the end of March 2021, almost 350,000 cases of coronavirus disease-2019 (COVID-19) have been confirmed in Ontario, Canada, with transmission rates continuing to increase rapidly across the province (Public Health Agency of Ontario, 2022). Windsor-Essex County was one of the hardest-hit regions in Ontario during the first and second waves of the COVID-19 pandemic, grappling with infection rates varying between the 1st and 4th highest in the province (Windsor-Essex County Health Unit, 2022). Due to their close contact caring for and treating patients with the disease, the risk of acquiring COVID-19 is significantly elevated among healthcare workers (HCWs; Lai et al., 2020; Tan et al., 2020; de Kock et al., 2021; Sun et al., 2021).

Historically, HCWs have assumed a critical role on the front lines of epidemics, facing increased workloads and risk of infection (Nickell, 2004). Previous research into the effects of the Sudden Acute Respiratory Syndrome (SARS), Ebola, H1N1, and COVID-19 outbreaks on HCWs found risk factors including the rapidly increasing number of cases, increased volume and intensity of workload, depletion of personal protective equipment, concerns about personal health and that of close contacts, and job-related stress all contributed to HCWs’ emotional burden (Chan-Yeung, 2004; Nickell, 2004; Muller et al., 2006; Ayanian, 2020; Lai et al., 2020). In particular, HCWs have reported high levels of psychological distress, anxiety, and social isolation during these crises (Maunder et al., 2003; Goulia et al., 2010; Lehmann et al., 2015; Lai et al., 2020). For some HCWs, symptoms of distress can persist even after an outbreak has subsided (Maunder et al., 2003).

In general, research into the COVID-19 pandemic has revealed that working on the frontlines is associated with an increased risk of adverse emotional outcomes, including emotional distress, anxiety, and depression (Al Maqbali et al., 2021; Sun et al., 2021). However, the risk of psychological distress is not restricted to those HCWs providing direct care to COVID-19 patients (Maunder et al., 2003). A growing number of studies have found no differences in levels of distress and the degree of worry between frontline and non-frontline staff, with high levels of burnout, insomnia, and anxiety experienced by hospital-based HCWs in general, regardless of contact with COVID-19 patients (Kang et al., 2020; Sahashi et al., 2020; Wu et al., 2020; Tiete et al., 2021).

Although most research has concentrated on the mental health of HCWs working in hospital settings, which significantly limits the generalizability of findings to other care settings (de Kock et al., 2021), there is some evidence that psychological distress is spread across all care settings (Al Maqbali et al., 2021). High rates of psychological distress have been found among primary care providers in China, and first responders have reported symptoms of anxiety and depression in the United States (Vujanovic et al., 2021; Zeng et al., 2021). Interviews conducted with Ontario HCWs working in the long-term care (LTC) and hospital sectors described similar experiences of intense distress, particularly about the lack of their organization’s instrumental protection and emotional support, which they felt left them vulnerable to infection and burnout (Brophy et al., 2020). Lessons learned from previous infectious disease outbreaks suggest that monitoring healthcare staff’s psychological distress is a critical step toward preventing personal exhaustion and reduced job performance (Lehmann et al., 2015). The purpose of this study is to describe and compare HCWs’ levels of psychological distress across care settings including first responder, primary care, hospital-based, LTC, and home-care health professionals in Windsor-Essex County, Ontario, Canada, during the first wave of the COVID-19 pandemic crisis. This study also investigates the risk factors associated with distress in HCWs overall and by care setting. Based on a previous research (e.g., Brophy et al., 2020), it was hypothesized that LTC and hospital-based HCWs would experience a higher rate of distress compared to HCWs employed at other settings and that distress scores would be influenced by risk factors such as age, gender, intensity of workload, risk of infection, concerns about personal health and that of close contacts, and job-related stress.

## Materials and methods

### Study design, setting, and participants

All clinical and non-clinical HCWs residing in Windsor-Essex County and actively employed at any healthcare setting, including hospitals, primary care clinics, first responder organizations, long-term care homes, and community-based health and social services, were invited to participate in the study. A two-wave repeated cross-sectional study design was used, which included web-based, self-administered questionnaires (Menard, 2002). Data presented in this report represent the first wave of data collection conducted from May 30^th^, 2020 until June 30th, 2020. A range of recruitment strategies were utilized to gain awareness of the study among our target population. Social media posts and paid Facebook advertisements were distributed with a link to an electronic Qualtrics survey. Targeted connections were made with communications staff at healthcare organizations within the community, including local hospitals, family health team clinics, the Windsor-Essex Medical Society, LTC and retirement homes, home-care providers, EMS, community health clinics, and local associations for allied health, chiropractor, and dental offices, requesting distribution of the study flyer and graphics on each organizations’ social media channels. A study notification was also posted on a local healthcare worker Facebook group. No incentives were offered to participate in this study. Participants provided informed consent prior to completing the 13-page study questionnaire. Respondents had the option to review responses on previous pages prior to submitting the survey. The study was cleared by the University of Windsor’s Research Ethics Board.

### Survey measures

The survey packaged consisted of 12 items measuring demographic and employment-related information, including healthcare setting, employment status, and risk of exposure to COVID-19 patients. Branching logic was utilized for the demographics and employment sections of the survey to conditionally display items based on previous responses to reduce the number of items on the survey. A total of 23 items adapted from previous research were included in order to understand perceptions about training, protection, and support provided by the healthcare organization during the outbreak and degree of concern participant’s had about daily work-related and personal sources of anxiety (Maunder et al., 2003; Nickell, 2004; Koh et al., 2005). Furthermore, a 5-item measure of job stress was used to assess daily work-related stress, with respondents rating their level of agreement of each item from strongly disagree to strongly agree (Koh et al., 2005). Items were then summed to calculate a total score. Perceptions of respect among healthcare teams were assessed using the 12-item Respectful Leadership Scale (van Quaquebeke and Eckloff, 2009). Items on this measure were rated on a 4-point scale, ranging from “never” to “always” and then summed to obtain a total score (van Quaquebeke and Eckloff, 2009). Higher scores reflect perceptions of high respect among members of the healthcare team. Finally, the 10-item Kessler Psychological Distress Scale (K10) was used to assess generalized psychological distress (Kessler et al., 2002). The K10 is a frequently employed and validated measure of distress that can be used as a diagnostic screening instrument for depression in the general Canadian population (Cairney et al., 2007). For this measure, respondents indicated how often they experienced negative feelings, such as nervousness, worthlessness, and depression, in the last 30 days. Responses were then coded using the scoring instructions recommended by Kessler and summed to create a total score (Kessler et al., 2002). Similar to previous studies, a clinically meaningful cutoff value of 16 or greater was used to identify the presence of high psychological distress (Maunder et al., 2003). A copy of the survey instrument is available upon request.

### Data analysis

All sociodemographic and occupational information, risk of exposure to COVID-19, COVID-19-related concerns, perceptions of training, protection, and support, job stress, respectful leadership, and psychological distress are described by measures of central tendency, including means (standard deviations) and medians (interquartile range (IQR)) for continuous variables and frequencies (percentages) for categorical variables, as appropriate. Differences between healthcare settings and psychological distress groups were assessed using univariate models, including independent Sample’s *t*-tests or one-way analysis of variable models for continuous variables (if data are normally distributed) or the Mann–Whitney U test or Kruskal–Wallis test (otherwise). Chi-square or Fisher’s exact tests were employed, as appropriate, for binary/categorical variables. All tests were two-sided, and a Bonferroni correction was used for each group of comparisons to adjust for family-wise error rate (Amstrong, 2014). Multivariate logistic regression analysis was used to assess the association between high and low psychological distress groups and potential risk factors based on the results of the bivariate analyses. The model was run using a backward stepwise selection algorithm, with variable contribution determined by the significance of the Wald statistic. Model assumptions were first tested and model fit assessed (Pregibon, 1981). Results were presented as adjusted odds ratios (OR), with corresponding 95% confidence intervals (95%CI).

## Results

### Demographic and employment characteristics

A total of 560 healthcare professionals working in any healthcare setting in Windsor-Essex County consented to participate in the study, of which 403 (72%) completed the full survey. Almost half of those who completed the survey were employed at hospitals (49.4%). Other healthcare settings represented included first responders (14.7%), primary care (7.9%), LTC (4.0%), home-care services (6.0%), and other community health and social services (18.4%; e.g., dental and chiropractic offices, pharmacies, and community health centers). Demographic and employment characteristics for the overall sample and by healthcare setting are found in Table 1. First response personnel were significantly more likely to identify as male (*p* = 0.000), and primary care and other community health and social service workers were significantly older (*p* = 0.000) compared to respondents from other healthcare settings. Community health and social service workers were less likely to identify as frontline employees and be employed at an organization directly involved in the care of COVID-19 patients.

**Table 1:** Sociodemographic and employment characteristics for overall overall sample and by healthcare setting.

Study characteristic	Overall % (*N* = 403)	Hospital % (*N* = 199)	First responders^a^ % (*n* = 58)	Primary care % (*n* = 32)	Long-term care % (*n* = 16)	Home care % (*n* = 24)	Other community health and social services^b^ % (*n* = 74)	*P*-value^c^^,^^d^
Gender Identity								0.000
Female	74.2	82.4	29.3	84.4	81.3	87.5	77	
Male	17.4	7.0	58.6	15.6	12.5	12.5	16.2	
Age								0.000
≤55 years	77.2	80.3	84.4	75	87.6	87.6	58.2	
≥55 years	15.8	10.6	12.1	25	6.3	12.5	32.5	
Marital Status								0.170
Married/common law	65.3	64.8	74.1	71.9	62.5	66.7	56.8	
Divorced/Separated/Window/er	9.4	8.5	8.5	21.9	6.3	16.7	10.9	
Single	17.1	17.6	12.1	6.3	25	12.5	24.3	
Ethnicity								0.748
White/Caucasian	80.4	80.4	79.3	78.1	81.3	83.3	81.1	
Other Ethnicities^e^	19.6	19.6	20.7	21.9	18.7	16.7	18.9	
Employment information								0.000
Full-time	77.7	73.4	93.1	90.6	43.8	58.3	85.1	
Part-time/casual	21.5	25.1	6.9	9.4	56.3	41.7	14.9	
Years working in healthcare								
Worked in healthcare <10 years	38.0	40.2	20.7	43.8	56.3	37.5	39.2	
Worked in healthcare >10 years	62.0	59.8	79.3	56.3	43.8	62.5	60.8	0.061
Exposure to COVID-19 patients								
Frontline employee	63.8	67.8	74.1	71.9	75	50	43.2	0.000
Non-frontline employee	33.3	27.6	24.1	28.1	12.5	50	55.4	
Organization of employment provides direct care to COVID-19 patients	67.5	80.9	94.8	31.3	56.3	70.8	27	0.000
Organization of employment does not provide direct care to COVID-19 patients	32.6	19.1	5.2	68.8	43.8	29.2	73	
Self-reported health								0.155
Poor or Fair	25.3	26.1	34.5	12.5	25	33.3	18.9	
Good or Excellent	71.5	69.9	62	87.5	75	66.7	77.1	

a First responders include emergency medical service personnel and firefighters.

b Other community and social services were identified as dental offices, chiropractic clinics, community mental health agencies, retirement homes, and assisted living.

c Chi-square analyses were used to test differences between care setting groups.

d Values of P < 0.006 (0.05/9) were considered statistically significant.

e Other ethnicities identified included Asian, Black, French Canadian, Indigenous, Metis, and Inuit.

### Professional and personal sources of anxiety about COVID-19

The degree of COVID-19-related concerns and worry perceived by survey respondents, overall and by healthcare setting, is described in Table 2. The most common source of anxiety reported by all respondents involved the risk of potentially transmitting COVID-19 to family members (72%). Concerns over managing higher workloads were significantly higher among LTC and home-care workers, and LTC workers reported significantly greater worry about having enough staff present to provide patient-care. Respondents working at other community health and social services were significantly more concerned about their family’s financial well-being compared to respondents from other healthcare settings.

**Table 2:** Degree of COVID-19-related concerns for overall sample and by healthcare setting.

	Overall % (*N* = 403)	Hospital % (*N* = 199)	First responders % (*n* = 58)	Primary care % (*n* = 32)	Long-term care % (*n* = 16)	Home care % (*n* = 24)	Other community health providers % (*n* = 74)	*P*-value^a^^,^^b^
Getting infected with COVID-19								0.627
Not at all	5.0	5	10.3		6.3	4.2	2.7	
Somewhat concerned	45.7	45.7	44.8	46.9	31.3	41.7	50	
Very to extremely concerned	49.1	48.7	44.8	53.1	62.5	54.2	47.3	
Infecting family members with COVID-19								0.805
Not at all	6.2	4.5	8.6	3.1	6.3	4.2	10.8	
Somewhat concerned	21.6	20.6	20.7	28.1	18.8	20.8	23	
Very to extremely concerned	72.0	74.9	70.7	68.8	75	70.8	66.2	
Being quarantined or forced to limit activities								0.131
Not at all	12.7	10.1	19	18.8	31.3	16.7	6.8	
Somewhat concerned	40.7	39.7	46.6	34.4	31.3	33.3	45.9	
Very to extremely concerned	46.2	49.7	34.5	46.9	37.5	45.8	47.3	
Ongoing work strain/over work due to COVID-19 pandemic								0.006
Not at all	12.2	10.1	15.5	6.3		16.7	18.9	
Somewhat concerned	32.0	30.2	29.3	46.9	12.5	16.7	41.9	
Very to extremely concerned	55.6	59.8	55.2	46.9	87.5	66.7	37.8	
Keeping yourself safe while on the job								0.077
Not at all	8.4	5.5	12.1	3.1	6.3	16.7	13.5	
Somewhat concerned	31.0	34.7	20.7	34.4	12.5	20.8	35.1	
Very to extremely concerned	60.0	58.8	67.2	62.5	81.3	62.5	51.4	
Your personal physical and mental health in general								0.009
Not at all	6.5	3	13.8	9.4	6.3	8.3	8.1	
Somewhat concerned	38.7	37.7	41.4	43.8	25	12.5	48.6	
Very to extremely concerned	54.6	58.8	44.8	46.9	68.8	79.2	43.2	
The financial well-being of yourself and/or your family								0.005
Not at all	25.6	24.1	46.6	21.9	31.3	12.5	17.6	
Somewhat concerned	37.7	42.2	19	46.9	31.3	45.8	35.1	
Very to extremely concerned	36.0	33.2	34.5	31.3	37.5	33.3	47.3	
Having enough staff to care for patients (0.000)								0.000
Not at all	22.8	19.1	31	21.9	0.0	8.3	36.5	
Somewhat concerned	30.3	30.7	34.5	34.4	6.3	29.2	29.7	
Very to extremely concerned	45.9	48.7	34.5	40.6	93.8	62.5	33.8	
Being able to balance working with taking care of yourself								0.024
Not at all	11.2	9.5	20.7	6.3	6.3	12.5	10.8	
Somewhat concerned	29.5	28.1	32.8	34.4	6.3	12.5	39.2	
Very to extremely concerned	59.3	62.3	46.6	59.4	87.5	75	50	

a Chi-square analyses were used to test differences between care setting groups.

b Values of P < 0.006 (0.05/9) were considered statistically significant.

### Occupational risk factors

No significant differences among respondents across healthcare settings were reported in perceptions about adequate access to personal protective equipment, precautionary measures, infection, prevention, and control procedures and training. However, participants working in LTC were significantly less likely to perceive that emotional supports were available to staff who needed them, whereas first responders were more likely to perceive that emotional supports were available (Table 3). Significant differences were observed between healthcare settings on job-related stress, such that workers from LTC experienced higher job stress, whereas primary care and other community health and social services reported lower job stress. First responders and home-care workers were more likely to perceive lower respect among their team members compared to HCWs from other care settings.

**Table 3:** Training, protection, and support reported by overall sample and by healthcare setting.

Training, protection, and support	Overall % (*N* = 403)	Hospital % (*N* = 199)	First responders % (*n* = 58)	Primary care % (*n* = 32)	Long-term care % (*n* = 16)	Home care % (*n* = 24)	Other community health providers % (*n* = 74)	*P*-value^a^^,^^b^
I had sufficient access to personal protective equipment								0.113
Not at all	8.9	7.0	1.7	21.9	12.5	8.8	12.2	
Somewhat sufficient	8.9	7.5	8.6	9.4	18.8	8.3	8.1	
Very sufficient	82.3	85.4	89.7	68.8	68.8	83.3	79.7	
The precautionary measures put in place were sufficient								0.423
Not at all	9.4	10.1	3.4	15.6	18.8	8.3	6.8	
Somewhat sufficient	10.4	10.1	10.3	3.1	18.8	8.3	13.5	
Very sufficient	79.7	79.4	86.2	81.3	62.5	83.3	78.4	
I had adequate information about COVID-19								0.887
Not at all	10.9	10.1	10.3	6.3	18.8	12.5	12.2	
Somewhat sufficient	11.2	9.5	13.8	9.4	18.8	8.3	13.5	
Very sufficient	77.8	79.9	75.9	84.4	62.5	79.2	74.3	
Infection control standards were adequately explained								0.513
Not at all	14.3	16.6	10.3	12.5	25.0	12.5	9.5	
Somewhat sufficient	13.8	11.1	12.1	18.8	12.5	25.0	16.2	
Very sufficient	71.9	72.4	77.6	68.8	62.5	62.5	74.3	
Infection control training was sufficient								0.284
Not at all	9.1	9.5	6.9	12.5	12.5	12.5	6.8	
Somewhat sufficient	21.4	24.1	13.8	18.8	43.8	20.8	18.9	
Very sufficient	68.8	65.3	79.3	68.8	37.5	66.7	74.3	
I feel that emotional support is available to those who need it								0.001
Not at all	24.7	10.6	8.6	18.8	50.0	16.7	17.6	
Somewhat sufficient	20.8	23.6	10.3	21.9	18.8	20.8	27.0	
Very sufficient	54.2	65.8	81.0	59.4	31.3	62.5	55.4	
Job stress, *M(SD)*	17.3 (4.11)	17.6 (3.80)	18.1 (4.20)	16.0 (4.45)	19.8 (2.67)	17.2 (4.29)	15.7 (4.37)	0.000
Median (IQR)	18 (15–20)	18 (15–21)	18.5 (15–21.5)	16 (14–18.75)	18.5 (18–23)	18 (15.5–19)	16 (13–18)	0.000
Respectful leadership, *M(SD)*	40.5 (14.02)	39.9 (14.28)	36.4 (14.47)	44.7 (13.73)	39.1 (15.89)	37.3 (13.48)	44.7 (11.65)	0.014
Median (IQR)	42 (30–53)	41 (28.5–52)	38 (26–50)	48.5 (36.5–55.75)	42 (24.5–50)	36 (28–47)	46.5 (38–55)	0.000

a Values of P < 0.006 (0.05/8) were considered statistically significant.

b Chi-square analyses, independent sample’s t-tests, or Kruskal Wallis tests were used, as appropriate, to assess differences between healthcare settings.

### Psychological distress

Of the 388 respondents who completed the K10 scale, 288 (74.2%) scored above the threshold of 16 or greater, reflecting the presence of high psychological distress. Differences between low and high psychological distress groups on study variables are illustrated in Table 4. Statistically significant differences in the presence of emotional distress were found between healthcare settings (*p* = 0.001), suggesting a greater proportion of the LTC and hospital groups (82.5 and 87.5%, respectively) reported experiencing greater symptoms of distress compared to primary care (68.8%), first responder (64.3%), and other community and social health groups (59.3%). Other risk factors significantly related to experiencing high psychological distress included identifying as female, being younger than 55 years old, being extremely concerned about managing workload and personal safety while working, personal physical and mental health, having enough staff to care for patients, balancing taking care of oneself with an increased workload, receiving adequate information and training on Infection Prevention and Control Canada (IPAC) procedures, higher job-related stress, and lower perceived respect among members of the healthcare team.

**Table 4:** Differences between low and high psychological distress groups on study variables.

	Low psychological distress group, (K10 < 16)%	High psychological distress group, (K10 ≥ 16)%	*P*-value^a^^,^^b^
Healthcare setting			0.001
Hospital (*N* = 189)	17.5%	82.5%	
First responders (*n* = 56)	35.7%	64.3%	
Primary care (*n* = 32)	31.2%	68.8%	
Long-term care (*n* = 16)	12.5%	87.5%	
Home care (*n* = 24)	25.0%	75.0%	
Other community health providers (*n* = 71)	40.8%	59.2%	
Gender Identity			0.000
Female	37.0	18.1	
Male	63.0	81.9	
Age			0.000
≤55 years	66.0	89.6	
≥55 years	34.0	10.4	
Marital Status			0.452
Married/common law	72.0	66.3	
Divorced/Separated/Window/er	10.0	9.7	
Single	12.0	19.8	
Employment status			0.487
Full-time	80.0	77.4	
Part-time/Casual	18.0	22.2	
Exposure to COVD-19 patients			
Frontline worker	38.0	31.3	0.357
Non-frontline worker	60.0	65.3	
Organization of employment provides direct care to COVID-19 patients	41.0	29.9	0.043
Organization of employment does not provide direct care to COVID-19 patients	59.0	70.1	
Getting infected with COVID-19			0.003
Not at all	8.0	3.8	
Somewhat concerned	57.0	42.0	
Very to extremely concerned	35.0	53.8	
Infecting family members with COVID-19			0.013
Not at all	11.0	4.5	
Somewhat concerned	26.0	19.4	
Very to extremely concerned	62.0	76.0	
Being quarantined or forced to limit activities			0.055
Not at all	17.0	11.8	
Somewhat concerned	47.0	38.2	
Very to extremely concerned	36.0	49.3	
Ongoing work strain/over work due to COVID-19 pandemic			0.000
Not at all	25.0	7.6	
Somewhat concerned	49.0	26.7	
Very to extremely concerned	26.0	65.3	
Keeping yourself safe while on the job			0.000
Not at all	16.0	5.6	
Somewhat concerned	39.0	28.5	
Very to extremely concerned	45.0	65.3	
Your personal physical and mental health in general			0.000
Not at all	22.0	1.0	
Somewhat concerned	62.0	31.3	
Very to extremely concerned	16.0	67.4	
The financial well-being of yourself and/or your family			0.003
Not at all	38.0	21.9	
Somewhat concerned	37.0	37.2	
Very to extremely concerned	25.0	39.9	
Having enough staff to care for patients			0.000
Not at all	35.0	18.8	
Somewhat concerned	33.0	28.1	
Very to extremely concerned	30.0	52.4	
Being able to balance working with taking care of yourself			0.000
Not at all	29.0	5.2	
Somewhat concerned	43.0	25.0	
Very to extremely concerned	28.0	69.8	
Training, protection, and support			
I had sufficient access to personal protective equipment			0.588
Not at all	11.0	8.3	
Somewhat sufficient	7.0	9.4	
Very sufficient	82.0	82.3	
The precautionary measures put in place were sufficient			0.355
Not at all	6.0	10.4	
Somewhat sufficient	9.0	10.8	
Very sufficient	84.0	78.5	
I had adequate information about COVID-19			0.036
Not at all	6.0	12.5	
Somewhat sufficient	7.0	12.8	
Very sufficient	87.0	74.3	
Infection, prevention, and control (IPAC) standards were adequately explained			0.000
Not at all	3.0	18.1	
Somewhat sufficient	10.0	14.9	
Very sufficient	87.0	67.0	
Infection control training was sufficient			0.000
Not at all	2.0	11.5	
Somewhat sufficient	13.0	24.3	
Very sufficient	84.0	63.5	
I feel that emotional support is available to those who need it			0.008
Not at all	5.0	17.4	
Somewhat sufficient	22.0	21.9	
Very sufficient	73.0	60.8	
Job stress, *M(SD)*	15.3 (4.32)	18.0 (3.79)	0.000
Median (IQR)	16 (13–18)	18 (16–21)	0.000
Respectful leadership, *M(SD)*	46.9 (11.80)	38.4 (14.0)	0.000
Median (IQR)	51 (40–55.5)	39 (28–50)	0.000

a values of P < 0.0021 (0.05/24) were considered statistically significant.

b Chi-square analyses, independent sample’s t-tests, or Kruskal Wallis tests were used, as appropriate, to assess differences between healthcare settings.

Logistic regression analyses revealed five factors significantly associated with the presence of high emotional distress among healthcare participants. In particular, greater odds of experiencing heightened psychological distress were found among HCWs who identified as female (OR: 3.25; 95%CI:1.65–6.58, *p* = 0.001), were younger than 55 years of age (OR: 3.57, 95%CI:1.64–7.76, *p* = 0.001), reported lower perceived respect among team members (OR.95; 95%CI: 0.926–0.973, *p* = 0.000), experienced greater concern about personal and mental health in general compared to some or no concern (OR: 9.36; 95%CI:4.55–19.26, *p* = 0.000), and greater concern about managing workload compared to some or no concern (OR: 2.18; 95%CI:1.13–4.20, *p* = 0.02).

## Discussion

HCWs play a vital role in the ongoing response to the COVID-19 crisis, which is expected to challenge their mental health and put them at a greater risk of experiencing distress symptoms than the general population (Kinman et al., 2020). Consistent with previous research on HCWs during the COVID-19 outbreak, more than 70% of HCWs in the current sample reported high emotional distress as measured by the K10, reflecting a significantly higher rate of distress found in recent international population studies (Kessler et al., 2002; Lai et al., 2020; Rahman et al., 2020; Elhessewi et al., 2021). In addition, heightened psychological distress was observed in over 50% of respondents from first responder, primary care, LTC, home-care, and other community health and social service groups, which is similar to recent findings of HCWs employed outside of hospital settings (Vujanovic et al., 2021; Zeng et al., 2021). Moreover, consistent with previous findings, no association was found between frontline and non-frontline workers’ experiences of emotional distress (Kang et al., 2020; Sahashi et al., 2020; Wu et al., 2020). Although, as hypothesized, high psychological distress was more likely for HCWs employed at hospitals and LTC homes compared to other care setting groups, these differences were indeed nullified after adjusting for other risk factors. These results underscore the importance for all healthcare organizations across the health system to support their staff’s well-being during the COVID-19 outbreak to reduce the risk of poor psychological outcomes among their workforce (Blake et al., 2020; Shanafelt et al., 2020).

The current findings also confirm that all HCWs are highly anxious about transmitting COVID-19 to their loved ones; however, in this study, their concerns were not highly related to emotional distress (Shechter et al., 2020; Walton et al., 2020). While some sources of anxiety were common among HCWs across care settings, such as being extremely worried about their safety while working and balancing personal care with the demands of the job, our findings indicate that other concerns may be amplified for HCWs at particular care settings. For example, LTC and home-care respondents were more anxious about managing higher workloads and having enough staff to provide patient care. In contrast, HCWs working at other community health and social agencies were more anxious about their family’s financial security, which may be due to significant changes in job roles and work environment as compared to pre-pandemic conditions (Greenberg, 2020). The sources of anxiety assessed in this study were informed by research on hospital-based HCWs; therefore, identifying COVID-related worries that may be unique to HCWs at different care settings is an important next step in developing tailored approaches to addressing staff concerns (Nickell, 2004; Shanafelt et al., 2020; de Kock et al., 2021).

Furthermore, consistent with our initial hypotheses, experiencing emotional distress varied by demographic, job-related, and psychological risk factors. In particular, those who were more likely to experience symptoms of emotional distress included HCWs who identified as female, those who were younger than 55, those who reported lower perceived respect among team members, and those that reported experiencing experienced greater worry about their physical and mental health and about managing their higher workloads, were more likely to experience symptoms of emotional distress. These findings suggest promising avenues for identifying those at higher risk of distress and developing effective intervention strategies that include the promotion of self-care (e.g., work breaks and healthy lifestyle behaviors) and the fostering of supportive and collaborative relationships among healthcare teams (Krasner, 2009; Miller et al., 2018).

The findings from this study should be considered in light of several limitations. First, the cross-sectional design limits the ability to draw conclusions about the longer-term psychological impact of the COVID-19 pandemic on HCWs. The relatively smaller sample size obtained in this study, particularly for HCWs from long-term and home-care sectors, and social media recruitment strategies may have resulted in response bias. The concerns and experiences of respondents may not be representative of other non-participating HCWs across different care settings and thus may limit the generalizability of these findings. In particular, a response rate could not be calculated for this study as information about the total population of HCWs employed in various healthcare settings in Windsor-Essex is not publically available. All measures used were self-report, and pre-existing mental health conditions were not assessed in this study, both of which may have resulted in inflated rates of psychological distress.

## Conclusion

Our findings add to the growing literature documenting the significant risk of adverse mental health outcomes for HCWs managing COVID-19 in first responder, hospital, primary care, LTC, home-care, and other community health and social service organizations. Our data were collected immediately following the peak of the first wave of the pandemic in Windsor-Essex County, a region that consistently reported higher infection rates than the Ontario provincial average. Follow-up investigations are warranted to understand the extent of the psychosocial impacts of COVID-19 on across healthcare workforce over time and to identify effective supports to promote emotional recovery across the healthcare system following the pandemic.

## Data availability statement

The raw data supporting the conclusions of this article will be made available by the authors upon reasonable request, without undue reservation.

## Ethics statement

The studies involving human participants were reviewed and approved by the Research Ethics Board at the University of Windsor (REB#20-085). The patients/participants provided their written informed consent to participate in this study.

## Author contributions

JV, LJ, and LM designed the study. JV drafted the manuscript and conducted the statistical analyses. All authors contributed to data collection, interpreted the study findings, revised the manuscript, approved the version to be published, and agreed to be accountable for all aspects of this work.

## Funding

This study was supported by a WE-SPARK Igniting Discovery Grants Program, Grant# 820618.

## Conflict of interest

The authors declare that the research was conducted in the absence of any commercial or financial relationships that could be construed as a potential conflict of interest.

## Publisher’s note

All claims expressed in this article are solely those of the authors and do not necessarily represent those of their affiliated organizations, or those of the publisher, the editors and the reviewers. Any product that may be evaluated in this article, or claim that may be made by its manufacturer, is not guaranteed or endorsed by the publisher.
